# Sex differences in the IQ-white matter microstructure relationship: A DTI study

**DOI:** 10.1016/j.bandc.2014.08.006

**Published:** 2014-11

**Authors:** Beate Dunst, Mathias Benedek, Karl Koschutnig, Emanuel Jauk, Aljoscha C. Neubauer

**Affiliations:** Department of Psychology, University of Graz, Austria

**Keywords:** Corpus callosum, DTI, Intelligence, Sex, TBSS

## Abstract

Sex differences in the relationship between general intelligence and brain structure are a topic of increasing research interest. Early studies focused mainly on gray and white matter differences using voxel-based morphometry, while more recent studies investigated neural fiber tracts using diffusion tensor imaging (DTI) to analyze the white matter microstructure. In this study we used tract-based spatial statistics (TBSS) on DTI to test how intelligence is associated with brain diffusion indices and to see whether this relationship differs between men and women. 63 Men and women divided into groups of lower and higher intelligence were selected. Whole-brain DTI scans were analyzed using TBSS calculating maps of fractional anisotropy (FA), radial diffusivity (RD), and axial diffusivity (AD). The results reveal that the white matter microstructure differs between individuals as a function of intelligence and sex. In men, higher intelligence was related to higher FA and lower RD in the corpus callosum. In women, in contrast, intelligence was not related to the white matter microstructure. The higher values of FA and lower values of RD suggest that intelligence is associated with higher myelination and/or a higher number of axons particularly in men. This microstructural difference in the corpus callosum may increase cognitive functioning by reducing inter-hemispheric transfer time and thus account for more efficient brain functioning in men.

## 1 Introduction

The investigation of how intelligence and sex differences are manifested in the brain’s structure has become an exciting research question in the differential psychological approach in the last decade. Although there are no sex differences in general intelligence, sex differences in the relationship between general intelligence and brain structure have been observed. One of the earliest reports goes back to Haier, Jung, Yeo, Head, and Alkire (2005). In an MRI study using voxel-based morphometry (VBM), they demonstrated that, in women, intelligence is positively related to white matter volume in the frontal lobe, whereas men show positive intelligence-gray matter correlations in frontal and parietal lobes. Thus, although the sexes do not differ in general intelligence, the neuroanatomical structures of intelligence are different for women and men. Burgaleta et al. (2012) tested the relationship between general intelligence and global brain features, like total and tissue-specific volumes, related to sex differences. Interestingly, their findings are not in line with Haier’s results. Women showed a positive intelligence-gray matter volume relationship but no significant intelligence-white matter volume correlation was found. For men, no significant correlations between general intelligence and total volumetric measures were observed. The discrepant findings could in part be the result from different analysis methods. While Haier et al. (2005) explored the relationship on a regional level, Burgaleta’s study analyzed total volumetric measures. These studies provide first evidence that the correlation between intelligence and the brain structure is moderated by sex.

While the focus of earlier studies lies mainly on volumetric differences using VBM, more recent studies investigated neural fiber tracts using diffusion tensor imaging (DTI) to analyze the white matter microstructure. Specifically, fractional anisotropy (FA), radial diffusivity (RD), and axial diffusivity (AD) provide estimates of the integrity and density of fibers and the degree of myelination.

Even though there exists no sex difference in general intelligence on a behavioral level, it becomes apparent from the literature reviewed above that the relationship between intelligence and brain structure varies between the sexes. Thus, the current study aims at testing whether the correlation between intelligence and the white matter microstructure using DTI differs between men and women.

### 1.1 Intelligence correlates of white matter microstructure

By now, few studies have employed DTI to characterize structural connectivity differences in the brain between lower and higher intelligent individuals. There is evidence that general intelligence is related to higher integrity of WM fiber tracts connecting parieto-frontal cortical areas (Barbey et al., 2013, Gläscher et al., 2010). This result is in line with the parieto-frontal integration theory, assuming that general intelligence is particularly associated with effective parieto-frontal information processing (Jung & Haier, 2007). Another study testing the relationship between intelligence and the white matter microstructure found positive correlations with FA in bilateral frontal and occipito-parietal regions (Schmithorst, Wilke, Dardzinski, & Holland, 2005), indicating higher white-matter fiber integrity of those regions in higher intelligent individuals. Clayden et al. (2012) demonstrated that FA in the splenium and left-side inferior longitudinal and arcuate fasciculi positively predicts intelligence. This tract connects regions within hemispheres, which is crucial for the integration of information between frontal (including Broca’s area) and temporo-parietal regions (including Wernicke’s area). Interhemispheric white matter microstructure differences between lower and higher intelligent individuals were found by Navas-Sánchez et al. (2014). They reported a positive correlation of intelligence with FA in the corpus callosum.

### 1.2 Sex differences in the white matter microstructure

Turning to sex differences in the white matter microstructure, Szeszko et al. (2003) reported that women have higher FA in the left frontal lobe as compared to men. Schmithorst, Holland, and Dardzinski (2008) found that females (average age of 12 years) show higher FA in the splenium of the corpus callosum, while males have higher FA in associative white matter regions (including the frontal lobes). Higher FA and lower RD in men as compared to women were reported by Menzler et al. (2011) in the corpus callosum, the cingulum, and the thalamus. While sex differences in the corpus callosum and cingulum have been previously observed (Westerhausen et al., 2003), the finding that men show higher thalamic FA accompanied by lower RD than women has not been described before. Instead, higher local efficiency in cortical anatomical networks was found in women, especially those with smaller brains, specifically in the precuneus, the precentral gyrus, and the lingual gyrus (Yan et al., 2011). Although these studies provide some evidence for sex differences in white matter structure, research on the intelligence-WM relationship has rarely considered sex a potential moderator variable.

### 1.3 Sex differences in the IQ-white matter microstructure relationship

Studies focusing on intelligence (Clayden et al., 2012, Schmithorst et al., 2005) typically apply statistical techniques to control for morphological differences associated with age and sex. According to Tang et al. (2010), sex differences in brain structure and function make it necessary to explore the relationship between intelligence and brain parameters separately for both sexes (even when there are no general ability differences in intelligence). Tang et al. (2010) analyzed intelligence differences separately for the two sexes and found that higher intelligent males show lower FA in the forceps major, while in females, FA in parts of the forceps major (extension of the splenium) is positively correlated with general intelligence. The negative FA correlation in men was interpreted as an indicator of interference from contralateral sides of the brain who rely mostly on the right side of the brain. The positive FA correlation in women was associated with the observation that the splenium may be larger in females.

A developmental study by Wang et al. (2012) used TBSS to study sex differences in the association between intelligence and white matter microstructure in the adolescent brain. Considering the whole sample, full-scale IQ was positively related to FA in the frontal part of the right inferior fronto-occipital fasciculus, which suggests that region specific increases in FA are associated with optimal cognitive performance. Moreover, in females, significant correlations between verbal IQ and FA could be found in two clusters including the left corticospinal tract and superior longitudinal fasciculus (a region associated with language). Considering full-scale IQ, however, no correlations with FA could be found neither in females nor males.

### 1.4 Research question

The literature usually reports no sex differences in general intelligence. From the above reviewed literature, however, it becomes evident that the relationship between intelligence and brain structure may vary between the sexes. Thus, the current study aims at testing whether sex moderates the correlation between intelligence and the white matter microstructure applying TBSS. Most of the research on white matter microstructure is based on region of interest (ROI) analyses or fiber tracking analyses. A novel method is to use tract-based spatial statistics (TBSS; Smith et al., 2006) to perform automated analysis of white matter integrity. TBSS uses a carefully tuned nonlinear registration method followed by a projection onto a mean FA skeleton. This skeleton represents the centers of all tracts common to the group and the resulting data fed into voxel-wise cross-subject statistics. Thus, TBSS combines the strength of both voxel-based and tractographic analyses to overcome the limitations of conventional methods including standard registration algorithms and spatial smoothing. TBSS is assumed to improve the sensitivity, objectivity, and interpretability of multi-subject diffusion imaging studies (Smith et al., 2006).

In addition to analyses of FA, we also investigate RD and AD, which allows for a clearer interpretation of potential FA differences in terms of myelination and axonal integrity. The FA measure represents the relative degree of anisotropy or directionality at each voxel and is an unspecific indicator of alterations in white matter microstructure (Peled, Gudbjartsson, Westin, Kikinis, & Jolesz, 1998). As FA is a summary measure of microstructural changes, it should be further characterized by RD and AD (Alexander et al., 2007, Alexander et al., 2011). RD indicates the diffusivity along directions which are orthogonal to the primary diffusion direction and is an indirect indicator of myelination (Song et al., 2005, Wu et al., 2011). In contrast, AD represents the diffusivity along the primary diffusion direction and is assumed to characterize the integrity of axons (Gao et al., 2009, Glenn et al., 2003, Sun et al., 2006). This study investigates sex differences in the relationship of intelligence and WM microstructure (FA, RD, AD) in an adult sample using TBSS.

## 2 Method

### 2.1 Participants

Participants were recruited via a local newspaper as well as the university’s mailing lists, to obtain a heterogeneous and not solely student sample. Participants had to be between 18 and 50 years old, speak German (mother tongue), and had to be without any neurological and/or mental disorders and medication. 16% of the participants had at least nine years of schooling, 60% had at least twelve years of schooling, and 24% had a university degree. Out of this screening pool of 298 participants who completed an intelligence structure test, 73 people (42 women and 31 men, aged between 18 and 50 years) were selected for this DTI study. Participants were selected on their *g*-factor score and represented individuals with relatively low average intelligence (IQ range 80–100) or relatively high average to superior intelligence (IQ range 110–130). Ten people were excluded from the analysis because of movement artifacts and technical acquisition problems during the MRI procedure. The final sample thus comprised 63 persons, who were divided into lower and higher intelligent women (*N*_WomenIQlow_ = 20 *N*_WomenIQhigh_ = 18) and men (*N*_MenIQlow_ = 12 *N*_MenIQhigh_ = 13) on the basis of their *g*-factor scores (see Table 1). All participants were right-handed and reported no medical or psychological disorders. Additionally, the MRI data were checked by an experienced radiological technical assistant and no abnormalities were detected. The participants gave written informed consent approved by the local ethics committee and received €15 for their participation in the study.

**Table 1:** Descriptive statistics of IQ scores and age.

		IQ high			IQ low			Total		
		*N*	*M*	*SD*	*N*	*M*	*SD*	*N*	*M*	*SD*
IQ	Female	18	122.98	9.90	20	93.09	5.34	38	107.25	16.98
	Male	13	122.36	9.58	12	91.91	6.77	25	107.74	17.55
	Total	31	122.72	9.61	32	92.65	5.84	63	107.45	17.07

Age	Female	18	25.58	9.45	20	34.60	11.01	38	30.33	11.14
	Male	13	27.24	7.36	12	40.19	11.64	25	33.46	11.52
	Total	31	26.28	8.54	32	36.70	11.40	63	31.57	11.31

### 2.2 Intelligence assessment

Participants’ general intelligence was assessed by means of the intelligence-structure-battery (INSBAT; Arendasy et al., 2008). The intelligence structure battery is a computerized adaptive intelligence test battery based on the Cattell-Horn-Carroll model (cf. McGrew, 2009), which is commonly used in German-speaking countries. In addition to psychometric *g*, the test battery assesses fluid intelligence (*G*_f_), crystalized intelligence (*G*_c_), quantitative knowledge (*G*_q_), visual processing (*G*_v_), short-term memory (*G*_stm_) and long-term memory (*G*_ltm_). Each of these second-order stratum factors can be measured by means of two or more subtests constructed by means of different approaches to automatic item generation (for an overview: Arendasy and Sommer, 2012, Arendasy and Sommer, 2013, Irvine and Kyllonen, 2002). All subtests were calibrated by means of the 1PL Rasch model and exhibited good construct and criterion validities (for an overview: Arendasy et al., 2008). In order to obtain a screening measure of psychometric *g* the following four subtests were completed: figural-inductive reasoning (FID), arithmetic flexibility (NF), verbal short-term memory (VEK) and word meaning (WB). The subtests were selected to cover a broad range of stratum two factors to avoid construct-underrepresentation in estimating psychometric *g* (cf. Major, Johnson, & Bouchard, 2011). All subtests were presented as computerized adaptive tests with a target reliability corresponding to *α* = .60. Factor loadings obtained with a representative Austrian norm sample were used to estimate the *g*-factor score based on the subtest results. The factor scores were further converted into IQ scores using the Austrian norm sample.

### 2.3 DTI acquisition

The DTI scans were collected on a 3-T Siemens Magnetom Skyra Scanner (Siemens Medical Systems, Erlangen, Germany), using a 32-channel head coil. A single shot echo planar imaging with a twice-refocused spin echo pulse sequence, optimized to minimize eddy current-induced image distortions (Reese, Heid, Weisskoff, & Wedeen, 2003) was performed on all subjects with the following parameters: TR/TE = 6600/95 ms, voxel size 2 × 2 × 2 mm, FOV = 240 mm, slices = 50, *b* = 1000 s/mm^2^, diffusion directions = 64. To minimize movement artefacts, the head of the subject was firmly fixed with cushions. All images were investigated to be free of motion, ghosting, high frequency and/or wrap-around artefacts at the time of image acquisition.

### 2.4 Diffusion tensor imaging (DTI) analyses

#### 2.4.1 Data preprocessing and analysis

Diffusion tensor imaging analysis was performed using FDT 3.0 (fMRIB’s Diffusion Toolbox V3.0) and TBSS (Tract-Based Spatial Statistics; Smith et al., 2006), part of FSL 5.0.6 (Smith et al., 2004). First, raw images were preprocessed using Eddy Current correction and a binary brain mask was created using BET (Brain Extraction Tool; Jenkinson, Pechaud, & Smith, 2005). Eigenvalues (*λ*1, *λ*2, *λ*3) and eigenvectors (*ε*1, *ε*2, *ε*3) of the diffusion tensor matrix for each voxel were computed from the DTI volumes for each subject on a voxel-by-voxel basis using established reconstruction methods (Basser & Jones, 2002). Thus, maps for fractional anisotropy (FA), axial diffusivity (AD = *λ*1), and radial diffusivity (RD = *λ*2 + *λ*3/2) could be generated to increase interpretability of our findings. All subjects’ FA data were then aligned into a common space using the nonlinear registration tool FNIRT (Andersson et al., 2007a, Andersson et al., 2007b), which uses a b-spline representation of the registration warp field (Rueckert et al., 1999). Next, the mean FA image was created and thinned to create a mean FA skeleton (template) which represents the centers of all tracts common to the group. A nonlinear registration, aligning all FA images to the high resolution FMRI58_FA image (target image) into 1 × 1 × 1 mm MNI152 standard space was chosen. The FA skeleton was thresholded at 0.20 to include major white matter pathways but avoid peripheral tracts (vulnerable to inter-subject variability). Each subject’s aligned FA data was then projected onto this skeleton and the resulting data fed into voxelwise cross-subject statistics. Furthermore, each subject’s aligned AD and RD data were projected onto the mean FA skeleton and the resulting data fed also into voxelwise cross-subject statistics. Prior to the voxel-wise analysis, we calculated the global mean values of each DTI index (FA, RD, and AD) from the whole- brain TBSS skeleton for each subject. To analyze the effect of IQ group and sex on global means of diffusion indices, three two-way ANCOVAs were computed with sex and IQ group as between-subjects variables and age as covariate.

#### 2.4.2 Voxel-based analysis

For the group analysis, we used the permutation tool “randomise” with 5000 permutations (Nichols & Holmes, 2002). The GLM includes both the effects tested (difference in FA between higher and lower intelligence groups, difference in FA between women and men and the two-way interaction intelligence group^∗^sex) and nuisance variables (age and global mean FA). Additionally, separate analyses for women and men testing differences in FA between higher and lower intelligent people corrected for age and global mean FA were done. The resulting statistical parameter maps were corrected for multiple comparisons by the family-wise error rate (FWE-corrected *p* < .05). Radial and axial diffusivity were compared using “randomise” in an analogous manner to the FA analysis.

The anatomical location of significant clusters was determined by the reference to the fiber tract-based atlas of human white matter (JHU ICBM-DTI81 White-Matter Labels, JHU White-Matter Tractography Atlas, Juelich Histological Atlas) implemented in FSL.

## 3 Results

### 3.1 Group differences in IQ

Descriptive statistics of the IQ scores and age are given in Table 1. In order to examine group differences in intelligence, a two-way ANOVA with sex and IQ group as between-subjects variables was computed. No significant differences were found between women and men and also the interaction of sex^∗^IQ group was not significant. The IQ groups differed significantly in general intelligence (*F*(1, 59) = 211.91, *p* < .001; *partial η*^2^ = .78). In order to examine group differences in age, a two-way ANOVA with sex and IQ group as between-subjects variables was computed. The analysis revealed that the less intelligent individuals are older than the more intelligent individuals (*F*(1, 59) = 17.96, *p* < .01; *partial η*^2^ = .23). There was neither a significant group difference for sex nor for the two-way interaction sex^∗^IQ group. Therefore, in all further analyses the effect of age was controlled statistically.

### 3.2 Global differences in the white mater microstructure

In a first step, we analyzed the effect of IQ group and sex on global means of diffusion indices (FA, RD, and AD) controlling for age. The analyses revealed that the IQ groups did not differ in global mean of FA, RD, and AD. There were neither significant group mean differences for IQ group (FA: *F*(1, 59) = .28, *ns*;. RD: *F*(1, 59) = .00, *ns*;. AD: *F*(1, 59) = 3.24, *ns*) nor for sex (FA: *F*(1, 59) = 1.50, *ns*;. RD: *F*(1, 59) = 2.45, *ns*; AD: *F*(1, 59) = 2.86, *ns*), nor a significant interaction (FA: *F*(1, 59) = .95, *ns*;. RD: *F*(1, 59) = .68, *ns;* AD: *F*(1, 59) = .22, *ns*).

### 3.3 TBSS whole brain analyses

#### 3.3.1 Fractional anisotropy

**Fig. 1:**
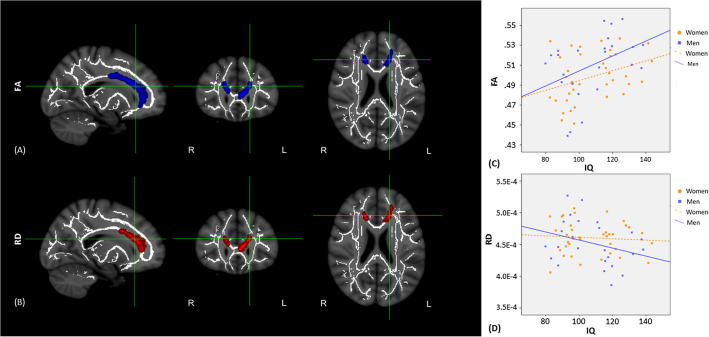
(A) Result of the whole-brain TBSS analysis comparing men of lower and higher IQ in their Fractional Anisotropy (FA). More intelligent men showed higher FA in the genu and body of the corpus callosum (*p* < .05 FWE-corrected). Blue means higher FA in more intelligent men. The figure is presented in radiological convention (*X* = 105, *Y* = 153, *Z* = 91). (B) TBSS result of the significant T-contrast between less and more intelligent men in Radial Diffusivity (RD) with a significant cluster in the corpus callosum (*p* < .05 FWE-corrected). Red means lower RD in more intelligent men. (C) Scatterplot depicting the correlation between intelligence and FA values in the significant cluster, separately for men (blue) and women (orange). (D) Scatterplot depicting the correlation between intelligence and RD values in the significant cluster, separately for men (blue) and women (orange). (For interpretation of the references to color in this figure legend, the reader is referred to the web version of this article.)

Explorative voxel-wise TBSS analyses of sex differences revealed no significant differences in FA values between women and men. A similar explorative analysis testing intelligence group differences and the two-way interaction IQ group^∗^sex was also not significant. In order to examine a potentially moderating effect of sex on the intelligence-FA relationship, analyses with the predictor intelligence were run separately for sex groups. The results indicated that less and more intelligent women did not differ in FA, but we discovered intelligence group differences for men in regional microstructural white matter. As shown in Fig. 1, more intelligent men showed higher FA compared to less intelligent men in the genu of the corpus callosum (CC) bilaterally and higher FA values in the body of the right CC relative to the global FA (*p* < .05, FWE corrected; see Table 2). In Table 3, mean as well as standard deviations for each group in each region are presented. Additionally effect sizes are reported.

**Table 2:** Clusters showing significant IQ group differences in men in fractional anisotropy (FA), radial diffusivity (RD), and axial diffusivity (AD). More intelligent men generally showed higher FA and lower RD, but no differences in AD.

	Voxel	*X*	*Y*	*Z*	*p*	Tract
FA	1113	105	153	91	.01	Genu of Corpus Callosum Left
	304	74	157	85	.03	Genu of Corpus Callosum Right
	70	75	122	106	.04	Body of Corpus Callosum Right

RD	893	105	153	91	.02	Genu of Corpus Callosum Left
	202	75	157	86	.04	Genu of Corpus Callosum Right
AD	–	–	–	–	–	–

**Table 3:** Descriptive statistics of fractional anisotropy (FA) and radial diffusivity (RD) for the significant clusters.

		IQ low		IQ high		*Cohen’s d*
		*M*	*SD*	*M*	*SD*	
*FA*						
Genu of the CC l	Female	.465	.029	.482	.020	0.68
	Male	.536	.029	.569	.033	1.06
Genu of the CC r	Female	.465	.029	.481	.029	0.55
	Male	.517	.041	.547	.042	0.72
Body of the CC r	Female	.501	.046	.513	.042	0.27
	Male	.512	.041	.565	.055	1.09
Total cluster	Female	.490	.028	.507	.020	0.67
	Male	.492	.032	.518	.030	1.00

*RD*						
Genu of the CC l	Female	.000420	.000027	.000417	.000023	−0.12
	Male	.000524	.000036	.000498	.000040	−0.68
Genu of the CC r	Female	.000457	.000030	.000451	.000019	−0.24
	Male	.000520	.000037	.000491	.000052	−0.64
Total cluster	Female	.000462	.000028	.000459	.000022	−0.12
	Male	.000469	.000022	.000447	.000041	−0.67

*Note.* CC = corpus callosum, l = left hemisphere, r = right hemisphere.

#### 3.3.2 Radial and axial diffusivity

Radial diffusivity, the potential marker of myelination, was lower in more intelligent men as compared to less intelligent men in the areas of altered FA in the genu of the CC bilaterally relative to the global RD (*p* < .05, FWE corrected, see Table 2). All other group comparisons (differences in RD between IQ groups, differences in RD between women and men, the interaction IQ group^∗^sex and differences in RD between less and more intelligent women) did not yield significant differences. Also, no significant effects emerged with respect to axial diffusivity, the potential marker of axonal integrity.

## 4 Discussion

This study aimed at examining sex and intelligence differences in the white matter microstructure. Our study was based on research demonstrating that the relationship of intelligence and brain structure may differ between the sexes (Tang et al., 2010), even when there are no general ability differences (Deary et al., 2007, Dykiert et al., 2009). In this study, the relationship of intelligence and WM microstructure was found to differ between the sexes: Intelligence-dependent white matter differences were only observed for men. Specifically, our analyses indicated that more intelligent men showed higher FA in the genu of the corpus callosum (CC) bilaterally and in the right body of the CC than less intelligent men. Additional analyses revealed that the higher FA in the genu of the CC was accompanied by lower RD in more intelligent males as compared to less intelligent males, but no significant differences in AD were observed.

### 4.1 Possible interpretations of differences in fractional anisotropy (FA) and radial diffusivity (RD)

The pattern of higher FA and lower RD observed here in absence of differences in AD in the genu of the CC could be interpreted either in terms of a higher axonal density or a higher degree of myelination (cf. Beaulieu, 2002, Jones et al., 2013). Higher axonal density, lower axonal caliber, as well as the higher degree of myelination should be reflected in lower RD and therefore higher FA (cf. Jones et al., 2013). Indeed, it has been demonstrated in eight different fiber tracts in mice that myelin loss alone (without axonal injury) can cause an increase in RD, while the AD remains unchanged (Song et al., 2002). Additionally, Song et al. (2005) evaluated the sensitivity of DTI parameters to detect the progression of myelin by testing demyelination and remyelination of corpus callosum in the mouse brain. Results demonstrated that radial diffusivity offers a specific assessment of demyelination and remyelination, as distinct from acute axonal damage. Thus, a more specific disruption of myelin is implied when an increase in RD occurs without an accompanying increase in AD (cf., Madden et al., 2012). However, the interpretation of RD as indicator of myelination is not straightforward and should be avoided especially in regions of complex tissue architecture (Sasson et al., 2010, Wheeler-Kingshott and Cercignani, 2009). We hence assume that the higher directionality of diffusion (as indicated by FA) is either due to differences in the number of axons and/or in the degree of myelination in more intelligent men.

Myelination of axons is known to increase the signal transmission speed (Waxman, 1977) and decrease the refractory time (time needed for repolarization before a new action potential can be supported by the axon; Felts et al., 1997, Sinha et al., 2006). Accordingly, the degree of myelination improves the integration of information across spatially distributed neural networks supporting cognitive and motor functions (Bartzokis et al., 2010, Fuster, 1999, Lu et al., 2011, Lu et al., 2013, Lutz et al., 2005, Mesulam, 2000, Srinivasan, 1999). The higher degree of myelination in more intelligent men thus might account for more efficient brain functioning (cf., Miller, 1994). The relationship of intelligence with the efficiency of brain functioning has been studied intensely throughout the past 20 years. It led to the postulation of the neural efficiency hypothesis assuming negative IQ-brain activation relationship, cf. Neubauer and Fink, 2009a, Neubauer and Fink, 2009b, Dunst et al., 2014). This relationship, however, can be moderated by other factors such as sex and task content (Dunst et al., 2013, Jaušovec and Jaušovec, 2008, Lipp et al., 2012, Neubauer et al., 2010, Neubauer et al., 2002, Neubauer et al., 2005). Males and females show the expected inverse IQ-brain activation relationship primarily in those tasks in which they usually perform better, i.e. males in visuo-spatial tasks and females in verbal and emotional intelligence tasks. However, in spite of the long research tradition, the functional and structural foundation of the neural efficiency phenomenon remains largely unclear. The findings of this study suggest that neural efficiency in men may be associated with more FA accompanied by lower RD (higher degree of myelination). Interestingly, Huster, Westerhausen, and Herrmann (2010) reported that interindividual variations in callosal morphology are associated with electrophysiological and behavioral performance measures. Large middle and posterior subregions of the CC were correlated with low reaction times and low stop-related P300. This is in line with our assumption that more FA in higher intelligent males may actually be associated with more efficient brain functioning by reducing inter-hemispheric transfer time. Although neural efficiency has been shown repeatedly when working on verbal tasks in the female brain (Neubauer et al., 2002, Neubauer et al., 2005), we observed no relationship between intelligence and white matter microstructure for females. Thus, efficient processing in women might be more related to gray matter differences (cf. Burgaleta et al., 2012). Gray matter (cell bodies, dendrites and short protrusions) is important for regional information processing (Gur et al., 1999). Yan et al. (2011) hypothesize that a higher percentage of GM in smaller brains increases the proportion of tissue available for computational processes, which further support high local network efficiency. This result was found for women by Yan et al. (2011).

### 4.2 Microstructural differences in the corpus callosum

The corpus callosum, together with the cingulum, the corticospinal tract, and the inferior fronto-occipital fasciculus, has been related to intelligence (Li et al., 2009). The corpus callosum, as the largest white matter tract in the human brain, plays an important role in higher cognition (cf. Hinkley et al., 2012). As the corpus callosum allows for functional interactions both within each hemisphere and between the two hemispheres, regions within the frontal, parietal, and occipital cortices that are implicated in cognitive domains are affected (Hinkley et al., 2012). Previous studies suggest that weakened integrity of the corpus callosum directly impairs cognitive function in aging adults (Voineskos et al., 2012, Zahr et al., 2009) whereas increased callosal thickness correlates positively with intelligence (Luders et al., 2007, Luders et al., 2011, Yu et al., 2008), processing speed (Penke et al., 2010), and problem solving abilities (van Eimeren, Niogi, McCandliss, Holloway, & Ansari, 2009). The corpus callosum, as part of the intelligence network (cf. Li et al., 2009), was found to differ between men and women with respect to white matter microstructure (Menzler et al., 2011). It was suggested that the differences between the sexes in the corpus callosum might be a microstructural correlate of the often reported sex differences in hemispheric lateralization (Menzler et al., 2011). Westerhausen et al. (2011) demonstrated that men show higher FA and lower diffusion strength compared to women in the genu and truncus of the corpus callosum. Interestingly, the diffusion parameters correlate with regional callosal size (exception: anterior genu subregions). The absolute size of the corpus callosum was found to be larger in men. As a larger corpus callosum might provide less noisy DTI measures, this may lead to an overall higher sensitivity in the analysis of intelligence-related differences in this structure in men.

### 4.3 Critical considerations and future directions

No significant AD differences between intelligence groups or women and men were observed in our study. As the axial diffusivity represents the diffusivity along the primary diffusion direction whereas the radial diffusivity indicates the diffusivity orthogonal to the primary diffusion direction (calculated by averaging the second and third eigenvalues of the diffusion tensor), it was hypothesized that axial diffusivity is an indirect indicator of the integrity of axons. Differences in FA and AD without differences in RD could be shown in studies investigating corpus callosotomy, optic neuritis, and axonal injury (Concha et al., 2006, Naismith et al., 2009, Song et al., 2003, Thuen et al., 2009). Thus, lowered FA driven by decreased AD is considered a marker of acute and primary axonal damage. Since our sample comprised healthy subjects who reported no medical or psychological disorders, we expected no differences in axial diffusivity related to intelligence. Bennett, Madden, Vaidya, Howard, and Howard (2010) suggested that this result pattern (lowered FA driven by decreased AD) may be also a consequence of disrupted macrostructural reorganization of the fibers, such as less coherent fiber alignment.

In this study, intelligence was associated with higher FA in the corpus callosum and lower radial diffusivity in men. The FA differences between lower and higher intelligent men were previously reported by Navas-Sánchez et al. (2014). In a similar vein, in the voxel-wise analysis male adolescents showed significant correlations between IQ and FA, mainly in the corpus callosum (genu, body and splenium). Interestingly, our findings are not in line with previous findings by Tang et al. (2010) or Wang et al. (2012). Tang et al. reported lower FA in the forceps major in highly intelligent males and higher FA in this region in highly intelligent females. The discrepant findings could in part be the result of the different analysis methods. While Tang et al. (2010) used a “multiple region brute-force” fiber tracking method before FA maps were analyzed using a region of interest approach, we analyzed whole-brain DTI scans without a priori hypotheses using TBSS calculating maps of FA, RD, and AD. Wang et al. (2012) did the same analyses as we did with the only difference that their sample comprised adolescents. They found no correlations between general intelligence and FA in separate sex analyses, but only a relationship between verbal IQ and FA in females. In part, this discrepancy might be related to age-related WM volume increases and age-related MTR (magnetization-transfer ration, indirect index of myelination) decreases during adolescence that was especially observed in boys but not in girls (Perrin et al., 2009). A limitation of this DTI study is that we are not able to directly image the degree of myelination in white matter (Alexander et al., 2011). Due to the effect of noise, the shape of the calculated diffusion ellipsoid and the pathology on the measured direction and magnitude of the eigenvalues and eigenvectors it is difficult to distinguish components of the microstructural pathology based on DTI indices alone. The major difficulty occurs in areas of low anisotropy such as gray matter, voxels affected by partial volume, areas of crossing fibers, or areas where the diffusion ellipsoid is oblate (cf. Wheeler-Kingshott & Cercignani, 2009). As morphological confounds affect primarily areas of low anisotropy, intelligence-related differences in the corpus callosum (high anisotropy) likely reflect true effects of intelligence on the white matter microstructure of men. Nevertheless, a replication of the present finding using complementary methods such as susceptibility tensor imaging (STI) or a longitudinal study comparing bundle-volume and configuration over time to uncouple true microstructural changes from morphological confounds (cf. Vos, Jones, Viergever, & Leemans, 2011), could be of particular interest. Also, future studies should try to match intelligence groups for age (rather than control effects of age statistically) and ensure equal sample sizes in all experimental groups. In this study fewer men were tested, thus the male group was slightly underpowered and the power to detect a two-way interaction when looking at sex and intelligence group is rather low. Finally, although our results are only partially consistent with prior findings, it should be acknowledged that this study, compared to previous relevant studies, used a comparably large sample as well as a more conservative threshold criterion (FWE corrected) which typically ensures robust findings.

## 5 Conclusions

The results provide evidence that white matter microstructure-correlates of intelligence are moderated by sex. By means of DTI-TBSS analyzes, the present study demonstrated that more intelligent men have higher FA accompanied by lower RD in the corpus callosum as compared to less intelligent men. According to this result and the given interpretation of FA and RD, intelligence might be associated with higher myelination and/or a higher axonal density in the tract connecting the right and left hemispheres and connecting areas within each hemisphere in men. Although further validation is still needed, our findings suggest that these microstructural differences may account for the more efficient brain functioning in more intelligent men. This finding may provide further insight into sex dimorphisms and underscores the importance of considering sex as an influential factor in neuroscience research.
